# The Gene Expression Profile of Milk Somatic Cells of Small Ruminant Lentivirus-Seropositive and -Seronegative Dairy Goats (*Capra hircus*) During Their First Lactation

**DOI:** 10.3390/v17070944

**Published:** 2025-07-03

**Authors:** Joanna Pławińska-Czarnak, Alicja Majewska, Joanna Magdalena Zarzyńska, Jarosław Kaba, Emilia Bagnicka

**Affiliations:** 1 Department of Food Hygiene and Public Health Protection, Institute of Veterinary Medicine, Warsaw University of Life Sciences, Nowoursynowska 159, 02-776 Warsaw, Poland; joanna_zarzynska@sggw.edu.pl; 2 Department of Physiology Sciences, Institute of Veterinary Medicine, Warsaw University of Life Sciences, Nowoursynowska 159, 02-776 Warsaw, Poland; alicja_majewska@sggw.edu.pl; 3 Division of Veterinary Epidemiology and Economics, Institute of Veterinary Medicine, Warsaw University of Life Sciences, Nowoursynowska 159, 02-776 Warsaw, Poland; jaroslaw_kaba@sggw.edu.pl; 4 Institute of Genetics and Animal Biotechnology, Polish Academy of Sciences, ul. Postepu 36A, Jastrzebiec, 05-552 Magdalenka, Poland; e.bagnicka@igbzpan.pl

**Keywords:** goat, milk somatic cells, small ruminant lentivirus, microarray, gene expression, *DUSP26*, *APBB2*, *PRLR*, *SCARA3*

## Abstract

Caprine arthritis and encephalitis (CAE), caused by small ruminant lentivirus (SRLV), is a key disease of goats, with chronic inflammation of joints and brain symptoms leading to losses in milk production and animal trade. In this study, we analyzed gene expressions in the milk somatic cells (MSCs) of seropositive (SRLV-SP) and seronegative (SRLV-SN) goats to identify transcriptomic changes using a non-invasive sampling method. Materials and Methods: This study was conducted on goats of two Polish breeds (Polish Improved White and Polish Improved Fawn), which were kept at the Institute of Genetics and Animal Biotechnology, Polish Academy of Sciences, during their first lactation. MSCs were isolated from milk, and gene expression was analyzed using the Goat Gene Expression Microarray. The results were verified by RT-qPCR for five genes (*DUSP26*, *PRLR*, *SCARA3*, *APBB2*, *OR4F4*). Statistical analysis was performed in GeneSpring 12 software. Results: Microarrays showed reduced expression of *DUSP26*, *PRLR*, *SCARA3*, *APBB2*, and *OR4F4* genes in SRLV-SP goats. RT-qPCR confirmed changes for *DUSP26*, *SCARA3*, and *APBB2*. Functional analysis indicated associations with immune processes and HIV-like pathways. Discussion: The results suggest that SRLV induces transcriptomic perturbations, especially in immunity-related genes. MSCs are an effective model for non-invasive studies, and further studies may support strategies for combating CAE.

## 1. Introduction

One of the most significant viral diseases affecting goats is caprine arthritis and encephalitis (CAE), which is caused by the small ruminant lentivirus (SRLV) [1]. SRLV, together with human, bovine, feline, equine, or simian immunodeficiency viruses (HIV, BIV, FIV, SIV, respectively), belongs to the *Retroviridae* family and genus *Lentivirus* and is a single-stranded RNA virus that leads to inflammation of the joints and brain in goats, manifesting as CAE, as well as Maedi-Visna Disease (MVD) in sheep [2]. SRLV shares several characteristics with human immunodeficiency viruses HIV-1 and HIV-2, particularly in its prolonged preclinical period, during which signs may not appear for months or even years, resulting in a chronic and often fatal illness. CAE and MVD are widespread globally among small ruminant populations, and they are listed by the World Organization for Animal Health (OIE) as two of the 117 notifiable terrestrial and aquatic animal diseases.

Among the various forms of the disease, the most common clinical signs include polyarthritis, wasting disease, and udder induration with hypogalactia [3].

In some European countries, there are voluntary CAE control programs that are heavily subsidized by governments. These programs involve removing clinically symptomatic and seropositive animals from herds and replacing them with healthy animals that have negative serological test results. However, this approach does not guarantee protection against SRLV infection. Despite the removal of some animals, the virus can still be present in the body of the animals, and antibodies may not appear in the blood for several months to 1.5 years after infection [4]. Furthermore, the interspecies transmission of the virus between sheep and goats contributed to the failure of the program in Switzerland. This occurred despite the considerable efforts made by breeders and the allocation of substantial state funding [5]. In this country and in northern Italy (Tyrol), the eradication program was initially established only for goats. After successfully eradicating genotype B of the virus, however, new infections emerged in goat herds [6,7]. In contrast, Norway implemented a comprehensive eradication program that addressed both scrapie-related lentivirus (SRLV) in goats and visna-maedi virus (VMV) in sheep. This program was effective in simultaneously combating these viruses along with other diseases affecting goats and sheep [8].

Due to the high genetic variability of the SRLV, there are currently no tests that are both highly sensitive and highly specific for detecting individuals at an early stage of infection. The occurrence of SRLV is most prevalent in industrialized countries, which is clearly related to the international movement of animals between herds of European dairy goat breeds [9,10]. Contradictory results on the influence of SRLV infection on the productivity of goats were stated [11]. Some studies indicated that CAE causes significant economic losses in the milk production of goats worldwide and difficulties in the sale of animals of high genetic value [12]. However, the papers indicated that the lack of SRLV infection on goat productivity can also be found [13]. Moreover, contradictory results were presented regarding immune system functioning depending on the serological status of animals [14]. Thus, detailed studies on gene expression connected with pathways involved in productivity and immunity are needed.

Animal research always poses a difficult dilemma regarding harming animals. Therefore, the use of milk somatic cells (MSCs) for transcriptomic studies is a very good alternative to biopsy or surgical tissue collection. It does not cause negative effects such as mastitis in goats and does not eliminate females from the herd after the experiment.

The aim of this study was to identify differences in the gene expression profiles in MSCs between dairy goats that are seropositive for small ruminant lentivirus (SRLV-SP) and those that are seronegative (SRLV-SN) during their first lactation. This was accomplished using a custom *Capra hircus* microarray. This study aimed to detect potential transcriptomic changes associated with SRLV infection that might impact immune response and productivity of goats. Additionally, it sought to evaluate the utility of MSCs as a non-invasive model for studying the pathogenesis of caprine arthritis and encephalitis (CAE).

## 2. Materials and Methods

### 2.1. Animals

This study used material from dairy goats of two breeds: Polish Improved White and Polish Improved Fawn (PWI and PFI, respectively). The animals were under constant veterinary supervision as a herd belonging to the Experimental Farm of the Institute of Genetics and Animal Biotechnology in Jastrzębiec (IGAB). The feeding system developed by the Institut National de la Recherche Agronomique (INRA) in France, according to which the goats were fed, was adopted by the Animal Research Institute in Poland [15]. The goats were fed corn silage with concentrates, calculated based on the average milk yield of the goats, and had access to water and hay ad libitum. In the summer, the diet was supplemented with green fodder by grazing on pastures. Two groups of goats at the climax of their first lactation were selected for this study: SRLV-seronegative goats (SRLV-SN; N = 4) and SRLV-seropositive goats (SRLV-SP; N = 4). The reference group consisted of seven SRLV-SN goats during their fourth lactation. All goats underwent a clinical examination, which included an assessment of the udder and milk. They were also tested using standard bacteriological culture methods to exclude subclinical intramammary infections before sampling. The SRLV-SN goats were housed separately from the SRLV-SP ones in separate enclosures with no contact between the two groups. SRLV-SN goats were milked first to prevent the risk of SRLV transmission through milking equipment. The goats were milked in a milking parlor equipped with a milking system dedicated to goats from DeLaval (Tumba, Sweden). After milking, all milking equipment was washed automatically. No invasive procedure was performed. Thus, no ethical permission was needed. After this study, the goats remained in the native herd in the Experimental Farms production system (IGAB).

### 2.2. Serodiagnosis of SRLV

In the IGAB herd, all goats have been tested for SRLV infection using ELISA tests for 20 years (regularly every six months). In the current experiment, two ELISA tests were used to test serum samples from animals selected for the study groups (ID Screen MVV/CAEV Indirect—Screening Test, ID.vet, Grabels, France, and IDEXX CAEV/MVV Total Ab Screening Test, IDEXX Laboratories, Inc., Westbrook, ME, USA) [16]. During the experiment, tests were also performed twice a year to identify new potential infections and eliminate infected animals from the reference and SRLV-SN groups. The laboratory analyses were performed after two years from collection, and the SRLV-SN group included only those that did not show seroconversion until the end of the second lactation. The SRLV-SP group included only those samples in which the presence of SRLV-SP was detected in the ELISA test at the age of 6 months, as well as in the subsequent tests conducted every six months. Regardless of serological status, all goats in this study were free from signs of CAE disease.

### 2.3. Milk Somatic Cells Sampling and RNA Isolation

Milk was collected during morning milking. All milk samples were tested for basic physicochemical parameters of milk (MilkoScan, FOSS, Hillerød, Denmark) and somatic cell count (IMCm, Bentley, Exton, PA, USA).

For transcriptomic analysis, RNA isolated from MSCs was used. MSCs were isolated according to the procedure described by Pławińska-Czarnak et al. (2019) for total RNA isolation from goat MSCs; the RNeasy Lipid Tissue Mini Kit (Qiagen, Hilden, Germany) was used according to the manufacturer’s procedure. Total RNA samples were stored at −80 °C until further analysis [17].

#### RNA Quality Assessment

NanoDrop (NanoDrop Technologies, Wilmington, DE, USA) and Agilent 2100 Bioanalyzer (Agilent Technologies, Santa Clara, CA, USA) were used to determine the quantity, purity, and integrity of RNA. RNA samples with RIN ≥ 7.5 were included for further analysis.

Basic information about the samples and data about their parameters were placed in a database specially created for this project and described by Pławińska-Czarnak et al., 2018 [18].

### 2.4. Microarray Analysis

The gene expression profile was determined in MSCs collected from SRLV-SN and SRLV-SP goats at the peak of their first lactation (N = 4 in each group). In this experiment, SurePrint G3 Goat Gene Expression Microarray, 8×60 K (Agilent Technologies, Santa Clara, CA, USA), and an Agilent Technologies Reagent Set (Low Input Quick Amp Labelling Kit, two color; RNA Spike-In Kit; Gene Expression Hybridization Kit; Gene Expression Wash Buffer Kit—Agilent Technologies, Santa Clara, CA, USA) were used. The reagents were used according to the manufacturer’s procedures.

Briefly, the experiment was performed using two-color microarrays. On each microarray, 300 ng of a cRNA Cy3-labeled common reference sample and 300 ng of a cRNA Cy5-labeled SRLV-SN goat or a SRLV-SP goat (each from a single goat) were hybridized simultaneously.

The common reference sample consists of equal amounts of RNA isolated from seven SRLV-SN goats during their fourth lactation. It is important to note that these RNA samples were not utilized as experimental samples in the microarray analysis.

Hybridization intensity analyses were conducted using an Agilent DNA Microarray Scanner G2505C. The data were extracted and background noise was subtracted using the standard procedures included in the Agilent Feature Extraction (FE) Software version 10.7.3.1. FE was also employed to perform Lowess normalization. A detailed description of the methodology is presented in the article Pławińska-Czarnak et al., 2021 [19].

### 2.5. Signal Detection and Statistical Analysis

The statistical analysis was conducted using GeneSpring 12 software (Agilent Technologies, Santa Clara, CA, USA). The significance of the differences was assessed using a moderated T-test, with a threshold of *p* < 0.05. To account for multiple testing, a Benjamini and Hochberg False Discovery Rate (FDR) of less than 0.5 was applied. The microarray data have been deposited in the Gene Expression Omnibus data repository under the identifier GSE299223.

### 2.6. Validation of Microarray Data

Reverse transcription quantitative PCR (RT-qPCR) was performed to validate the findings from the microarray analysis. We confirmed the expression levels of five genes: dual-specificity phosphatase 26 (putative) (*DUSP26*), prolactin receptor (*PRL-R*), scavenger receptor class A, member 3 (*SCARA3*), amyloid beta (A4) precursor protein-binding family B, member 2 (*APBB2*), and olfactory receptor *OR4F4*, all from *Capra hircus*. The sequences for these genes, as well as the reference gene, *peptidylprolyl isomerase A *(*cyclophilin A*, *PPIA*), were obtained from the Ensembl or NCBI databases. Primers were designed using Primer-Blast software (Primer-BLAST software (NCBI, Bethesda, MD, USA; version 1.0.5)) and were verified using Oligo Calc: Oligonucleotide Properties Calculator to ensure they did not exhibit self-complementarity. Additionally, the secondary structures of the amplicons were analyzed using the m-fold web server [20,21]. The two reference genes selected for this study were *18S ribosomal RNA* (*18S rRNA*), which is involved in protein synthesis, and *ribosomal protein large*, *P0 *(*RPLP0*). These genes were chosen based on results from previous studies [22]. To validate the stability of the reference genes, we used the GeNorm and NormFinder programs. All primer sequences are listed in Table 1.

The RT-qPCR reactions were performed in triplicate using SYBR Select Master Mix (Applied Biosystems, Waltham, MA, USA) on a Stratagene M×3005P Quantitative PCR instrument (Agilent Technologies, Waldbronn, Germany), following the manufacturer’s protocol. The reaction conditions included an initial polymerase activation at 95 °C for two minutes, followed by 40 amplification cycles. Each cycle consisted of denaturation at 95 °C for 15 s, annealing at 58 °C for 15 s, and extension at 72 °C for one minute.

The relative expression of the target gene was quantified as the mean of triplicate measurements for each biological sample. Results were calculated using the 2^−ΔΔCT^ method [23]. Two reference genes were used for normalization; their geometric mean CT was applied to account for potential variability in expression of the target genes of interest and to improve the accuracy of normalization.

### 2.7. Functional Analysis of Differentially Expressed Genes

To clarify the biological significance of the differentially expressed genes (*DUSP26*, *APBB2*, *SCARA3*, *PRLR*, *OR4F4*) in MSCs of goats that are seropositive for (SRLV-SP) compared to seronegative goats (SRLV-SN), we conducted a functional analysis. This analysis utilized Pathway Studio 11, along with DAVID (Database for Annotation, Visualization, and Integrated Discovery) and STRING (Search Tool for the Retrieval of Interacting Genes/Proteins). These tools were used to identify signaling pathways and molecular interactions associated with genes, proteins, and cells, specifically focusing on the mitogen-activated protein kinase (MAPK) pathway, immune response, and oxidative stress.

## 3. Results

### 3.1. Milk Yield and Composition in Relation to SRLV Serological Status

The characteristics of milk yield and its basic composition in morning milking in both studied groups are presented in Table 2. The arithmetic mean of MSCs was higher in the SRLV-SP group compared to the SRLV-SN group, with very similar standard deviations (SDs). In contrast, the SRLV-SN goats exhibited greater values for milk yield, fat content, protein content, and casein content; however, they had higher SD for each trait. Meanwhile, the lactose concentration remained similar between the two groups; however, they had higher SD, thus a higher coefficient of variation than for other traits (CV).

### 3.2. Microarray Analysis, Along with Validation Through Reverse Transcription Quantitative PCR (RT-qPCR)

Three reference genes were evaluated for their expression stability in milk somatic cells (MSCs) of dairy goats: peptidylprolyl isomerase A (PPIA, also known as cyclophilin A), ribosomal protein large P0 (RPLP0), and 18S ribosomal RNA (18S rRNA). Gene stability analysis was performed using the NormFinder algorithm implemented in GenEx software https://multid.se/genex/, accessed on 27 June 2025 (MultiD Analyses AB, Gothenburg, Sweden). Among the tested candidates, PPIA and 18S rRNA were identified as the most stably expressed genes across all experimental samples. Therefore, the geometric mean of PPIA and 18S rRNA expression levels was used for normalization of target gene expression data (Supplementary Materials).

The results of the microarray analysis showed significant changes in the expression of only five genes: *DUSP26*, *APBB2*, *PRLR*, *SCARA3*, and *OR4F4*, which showed lower expression in MSC SRLV-SP goats than in SRLV-SN goats. Validation using RT-qPCR confirmed consistent downregulation for three genes (*DUSP26*, *APBB2*, and *SCARA3*). In contrast, no statistically significant change in expression was observed for PRLR, although the trend remained consistent. For OR4F4, amplification was either undetectable or occurred only after the 42nd cycle of the RT-qPCR assay, indicating insufficient or unreliable transcript detection. The results are summarized in Table 3. Microarray analysis revealed a significant downregulation of *DUSP26* in the SRLV-SP group (−11.38-fold, *p* = 4.61 × 10^−5^), which was confirmed by RT-qPCR (−3.92-fold, *p* = 0.01939). Similarly, *SCARA3* was significantly downregulated in SRLV-SP goats (−3.56-fold, *p* = 8.15 × 10^−5^ in microarray; −3.39-fold, *p* = 0.02875 in RT-qPCR). In addition, *APBB2* was downregulated in SRLV-SP goats (−2.97-fold, *p* = 5.49 × 10^−5^ in microarray; −1.77-fold, *p* = 0.039 in RT-qPCR).

**Figure 1:**
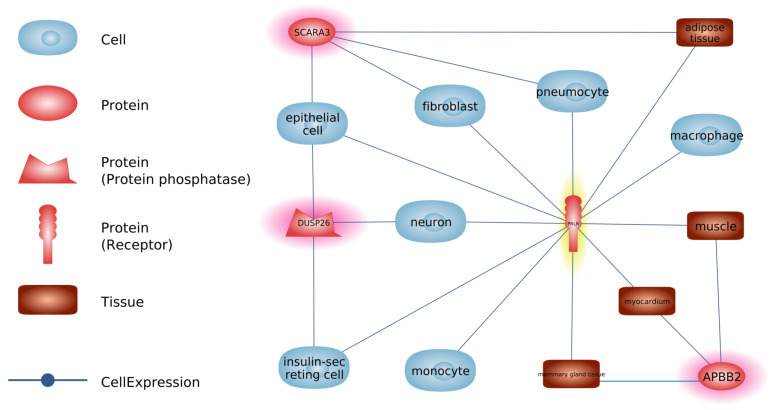
The connections between specific genes downregulated in MSCs of SRLV-SP goats and particular tissues and cells. *DUSP26—Capra hircus* dual-specificity phosphatase 26; *PRLR—Capra hircus* prolactin receptor; *SCARA3—Capra hircus* scavenger receptor class A, member 3; *APBB2—Capra hircus* amyloid beta (A4) precursor protein-binding, family B, member 2. Genes exhibiting a statistically significant decrease in expression are marked in red, while the gene showing non-significant downregulation in MSCs from SRLV-seropositive goats is marked in yellow.

The activities of *DUSP26*, *SCARA3*, and *APBB2* genes were confirmed in cells and tissues, which are simultaneously the target organs of SRLV; the virus can modulate the immune response and cellular processes in the targeted cells and tissues and cause pathological changes in them (Figure 1).

**Figure 2:**
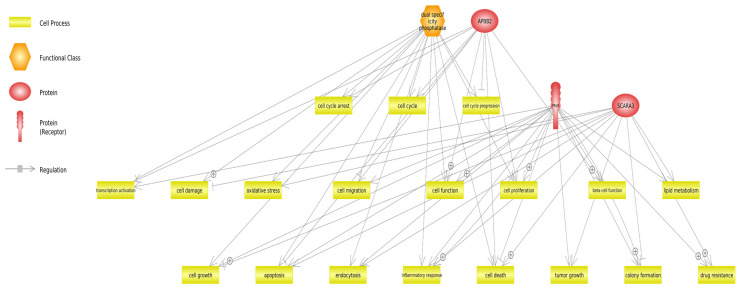
Functional classification of proteins involved in cellular processes. The proteins DUSP26 (dual-specificity phosphatase 26), APBB2 (amyloid beta precursor protein-binding family B, member 2), SCARA3 (scavenger receptor class A, member 3), and the prolactin receptor (PRLR) are involved in several interconnected cellular processes that are critical for maintaining cellular homeostasis and responding to environmental signals. DUSP26 plays a significant role in the regulation of the cell cycle, apoptosis, and tumorigenesis. Its activity is modulated by the signal transduction pathway initiated by growth factors. Similarly, APBB2 is involved in the regulation of cell migration, cellular function, intracellular trafficking, tumorigenesis, and chemoresistance. APBB2 is regulated by both PRLR and growth factor-mediated signal transduction pathways. SCARA3 contributes to the regulation of tumorigenesis, chemoresistance, cell adhesion, and lipid metabolism. The PRLR, in turn, regulates the expression of APBB2 and is itself involved in the control of the cell cycle, DNA replication, apoptosis, and cell death. PRLR is also regulated by growth factor signaling pathways. Collectively, these proteins participate in essential processes related to cell proliferation, survival, and the response to various stressors, including oxidative and oncogenic stress.

The analyzed genes are involved in overlapping cellular processes, suggesting potential functional convergence. Specifically, both *DUSP26* and *PRLR* are implicated in pathways related to apoptosis and the cell cycle, indicating roles in the regulation of cell survival and proliferation. In contrast, *APBB2* and *SCARA3* are associated with chemoresistance, which may reflect their involvement in cellular stress responses and mechanisms of drug resistance. The association of DUSP26, APBB2, and SCARA3 with tumorigenesis-related pathways may indicate virus-induced dysregulation of cellular functions that overlap with those observed in neoplastic processes, particularly in the context of chronic infection and inflammation. Notably, *DUSP26*, *ARRB2*, and *PRLR* are all regulated through growth factor-mediated signal transduction pathways, underscoring their roles in cellular responses to external proliferative and differentiation signals. Figure 2 illustrates the cellular processes in which proteins encoded by the *DUSP26*, *SCARA3*, *APBB2*, and *PRLR* genes are involved.

Functional analysis of downregulated genes in our study revealed that the protein products of DUSP26, SCARA3, APBB2, and PRLR positively regulate many cell processes such as transcription activation, cell migration, cell proliferation, lipid metabolism, and many others (Figure 2), wherein the inflammatory response, cell death, and colony formation are regulated by all three proteins.

## 4. Discussion

The mammary gland is a dynamic organ whose development and function depend on tightly regulated cellular processes, including proliferation, differentiation, and immune responses [24].

One of three genes that we found to be downregulated in the MSCs of SRLV-seropositive goats was *DUSP26*, which encodes a dual-specificity phosphatase that negatively regulates MAPK pathways, including ERK1/2, JNK (MAPK8), and p38, which are critical for cellular responses to stress, inflammation, and apoptosis [25]. *SCARA3* encodes a class A scavenger receptor, predominantly expressed in macrophages and dendritic cells, and plays a key role in pathogen recognition, phagocytosis, and the mitigation of oxidative stress [26]. *APBB2* encodes a protein that interacts with the cytoplasmic domain of the amyloid precursor protein (APP) and is involved in cellular signaling, differentiation, and possibly in mammary gland development.

Furthermore, *DUSP26* appears to participate in the regulation of immune and inflammatory responses. Notably, *DUSP26* has also been implicated in amyloid metabolism. Under hypoxic conditions, it enhances the generation of amyloid-β (Aβ42) by promoting the axonal transport of APP through a JNK-dependent mechanism, which alters the subcellular localization of the γ-secretase complex [27].

Viral infections, such as those caused by small ruminant lentivirus (SRLV), can disrupt these processes, leading to altered gene expression and impaired tissue homeostasis. Understanding the molecular mechanisms underlying these changes is crucial for identifying potential biomarkers and therapeutic targets.

While direct studies on the effect of APBB2 on cell migration are limited, there is evidence suggesting that Fe65 family proteins, which include APBB2, play a role in regulating cytoskeletal dynamics and cell migration. For example, the adaptor proteins NCK1 and NCK2, which are involved in cell signaling, have been shown to regulate cell proliferation and actin dynamics in various tissue types. This may indicate that APBB2 operates through similar mechanisms [28]. The NF-κB family regulates inflammation and cell survival. Low APBB2 expression may disrupt NF-κB, causing excessive cytokine production (e.g., IL-1β, TNF-α) [27], which in SRLV infections may promote immunosuppression or chronic inflammation, aiding viral persistence.

**Figure 3:**
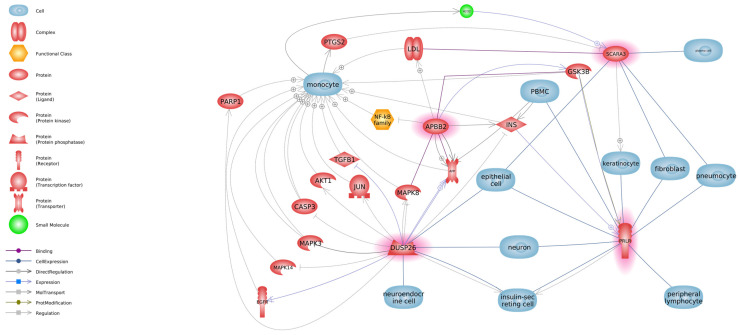
Network of molecular interactions linking DUSP26, SCARA3, APBB2, and PRLR with inflammation, oxidative stress, and signaling pathways. Genes marked in red shadow showed decreased expression in SRLV-SP goats relative to SRLV-SN goats.

We have demonstrated altered expression of only three genes out of more than 25K studied—*DUSP26*, *SCARA3*, and *APBB2*—in MSCs of goats infected with SRLV. To better understand their possible roles in inflammation, oxidative stress, and cell signaling pathways relevant to mammary gland physiology and SRLV pathogenesis, we propose a model illustrating the putative molecular interactions between these genes, cellular components, and associated proteins (see Figure 3).

Protein–protein interaction analysis using STRING suggests potential associations between DUSP26 and several MAPKs, including MAPK8, MAPK1, and MAPK3, with intermediate-to-high confidence scores (≥0.7) [29] (Figure 3). DUSP26 has been hypothesized to influence cell adhesion, and indirect interactions with kinesin motor proteins such as KIF3—key mediators of N-cadherin trafficking—may be involved, although this requires further investigation [30]. SCARA3 may play a protective role in oxidative stress responses in epithelial cells, including keratinocytes, although direct regulation remains to be clarified. APBB2 is known to interact with amyloid precursor protein (APP), influencing its processing and signaling. Although no direct interaction between DUSP26 and APP has been experimentally confirmed, indirect functional links may exist through the regulation of MAPK signaling pathways. DUSP26 negatively regulates kinases such as MAPK8 (JNK), which are involved in the phosphorylation and processing of APP, suggesting a possible indirect influence on APP-related cellular mechanisms.

SCARA3 functions as a cellular defense mechanism against oxidative stress by scavenging reactive oxygen species (ROS) and their harmful byproducts. Under physiological conditions, SCARA3 expression is upregulated in response to mild oxidative stimuli, such as UV radiation and hydrogen peroxide, thereby mitigating oxidative damage. However, excessive oxidative stress can lead to the downregulation of SCARA3 expression, compromising the cell’s antioxidant defense system. The diminished expression of SCARA3 results in the accumulation of ROS, which can activate various pro-inflammatory signaling pathways. One such pathway involves the upregulation of PTGS2 (Prostaglandin-Endoperoxide Synthase 2), also known as COX-2, a key enzyme in prostaglandin biosynthesis that plays a pivotal role in mediating inflammation. Elevated ROS levels can induce PTGS2 expression, thereby amplifying the inflammatory response. The downregulation of SCARA3 exacerbates oxidative stress, which in turn upregulates PTGS2 expression, leading to enhanced inflammatory responses. This interplay underscores the critical role of SCARA3 in modulating oxidative stress and inflammation [31]. Low APBB2 expression may disrupt cellular homeostasis, promoting lipid accumulation in neurodegenerative diseases like Alzheimer’s disease (AD) [32]. Dysregulated LDLR (Low-Density Lipoprotein Receptor), involved in cholesterol transport, may enhance oxidative stress, which is linked to reduced *APBB2* activity in APP pathways [33]. GSK3B encodes a kinase that phosphorylates APP, driving neurodegeneration. Low *APBB2* expression may increase GSK3β activity, elevating APP phosphorylation and amyloid-β accumulation, characteristic of AD [34]. MAPK8 encodes JNK, mediating stress and apoptosis. Low *APBB2* expression may boost JNK activity, worsening oxidative stress and apoptosis in neurons and immune cells, potentially weakening immune responses in infections like SRLV [35,36]. The NF-κB family regulates inflammation and cell survival. Low APBB2 expression may disrupt NF-κB, causing excessive cytokine production (e.g., IL-1β, TNF-α), which in SRLV infections may promote immunosuppression or chronic inflammation, aiding viral persistence [37,38].

DUSP26 also appears to play a role in the regulation of immune and inflammatory responses. Moreover, DUSP26 has been implicated in amyloid metabolism. Under hypoxic conditions, it enhances the generation of amyloid-β (Aβ42) by promoting the axonal transport of APP through a JNK-dependent mechanism that alters the subcellular localization of the γ-secretase complex [39].

### 4.1. Downregulation of DUSP 26 and APBB2 and Potential Connections with MSCs in SRLV-SP Goats

In goats infected with SRLV, reduced expression of *DUSP26*, encoding a dual-specificity phosphatase, may result in heightened MAPK signalling, potentially exacerbating inflammatory responses and tissue damage. This proposed mechanism resembles that observed in HIV infection, where MAPK dysregulation contributes to immune dysfunction [40]. The MAPK pathway, encompassing cascades such as ERK1/2, JNK, and p38, is a critical regulator of cellular responses to external stimuli, including viral infections, by modulating gene expression, cell proliferation, and apoptosis.

Both *DUSP26* and *APBB2* were downregulated in the MSCs of SRLV-SP goats, suggesting a potential co-regulation in response to viral infection and inflammation.

*DUSP26* is involved in regulating MAPK signaling and protecting cells from oxidative stress and apoptosis, primarily by modulating pathways such as ERK1/2, JNK (MAPK8), and p38. Its mitochondrial localization also links it directly to metabolic homeostasis and ROS control.

*APBB2*, on the other hand, encodes a protein that interacts with the intracellular domain of the amyloid precursor protein (APP), influencing APP processing and intracellular signaling, and has been implicated in neuronal differentiation and possibly mammary gland development.

Although a direct interaction between DUSP26 and APBB2 has not been established, both proteins converge functionally on stress-response pathways and APP-associated signaling.

Notably, APP processing and transport have been shown to be influenced by JNK activity—a pathway negatively regulated by DUSP26. Thus, reduced expression of both *DUSP26* and *APBB2* could potentially amplify dysregulation in APP trafficking, oxidative stress, and cell death signaling in response to viral infection or hypoxia-related stress.

This functional convergence suggests that their co-downregulation may disturb cellular homeostasis through combined impairments in stress adaptation, mitochondrial function, and APP-related signaling.

### 4.2. Downregulation of SCARA3

Downregulation of *SCARA3* was also observed in the same group. SCARA3 is involved in the regulation of immune responses and the maintenance of cellular homeostasis. It is expressed in various immune cells, including macrophages and dendritic cells, where it contributes to innate immunity by recognizing and clearing pathogens such as bacteria and viruses. This function is particularly relevant in the context of viral infections, including those resembling the hepatitis C virus (HCV) in hepatocellular carcinoma (HCC) [41]. In addition to pathogen recognition, SCARA3 facilitates the phagocytosis of apoptotic cells and cellular debris by macrophages, a process essential for immune surveillance and tissue maintenance. This phagocytic activity ensures the clearance of dying cells, preventing the release of potentially harmful intracellular contents and maintaining tissue integrity. As a class A scavenger receptor predominantly expressed in innate immune cells, SCARA3 contributes to host defense by facilitating pathogen recognition and phagocytosis. SCARA3 also plays a protective role against oxidative stress, which is a critical factor in the progression of various diseases, including multiple myeloma and other cancers. By mitigating oxidative damage within the cellular microenvironment, SCARA3 supports cell survival and contributes to therapy resistance in these pathological conditions [42,43,44].

### 4.3. Low Expression of APBB2

Expression of the next gene, *APBB2*, was reduced in SRLV-SP animals, as well. The *APBB2* gene product interacts with the cytoplasmic domain of APP and has been implicated in intracellular signaling pathways associated with differentiation and possibly mammary gland development. Thus, the *APBB2* gene plays a significant role in APP processing and Alzheimer’s disease pathogenesis [45]. Activity of γ-secretase, which is involved in APP processing, has also been associated with mammary epithelial cell differentiation, suggesting a potential mechanistic link to mammary gland development [46]. Moreover, γ-secretase is involved in other cellular processes, including Notch signaling and cell cycle regulation [47]. While *APBB2* is primarily studied in the context of neurodegenerative diseases, recent studies suggest potential roles in other tissues, including mammary gland development, due to its involvement in cellular differentiation and signaling pathways [48]. Low expression of APBB2 may induce a cascade of alterations in signaling pathways involving LDL, GSK3B, INS, APP, MAPK8, and the NF-κB family, affecting immune responses, cellular metabolism, and inflammation.

Although SRLV is not classified as an oncogenic virus, the association of DUSP26, APBB2, and SCARA3 with tumorigenesis-related pathways may reflect virus-induced dysregulation of cellular processes such as proliferation, stress response, and immune modulation, which are also involved in tumor biology.

### 4.4. Association of Studied Gene Expressions with Milk Yield and Composition

The arithmetic mean values of milk yield and its basic component contents were more or less similar in both groups and did not differ from the values presented for these breeds for the entire Polish population. SD of studied traits were much lower in SRLV-Sp than for SRLV-SN goats for composition milk traits, but still the coefficient of variations (CVs) were under 20%. The highest variation was noted for MSCs; however, it should be stressed that MSCs were in the range typical for goat milk from healthy goats, i.e., below 1 × 10^6^ in both groups [49,50].

The results of this study may indicate that reduced expression of *DUSP26* in SRLV-SP goats leads to increased activity in the MAPK pathways, particularly JNK and p38. This heightened activity contributes to stress-induced apoptosis and inflammation in the mammary gland cells. This finding is supported by a higher somatic cell count and lower milk yield in SRLV-SP goats. A study by Patterson et al. (2012) demonstrated that DUSP26 inhibits p38 signaling in neuronal cells, which helps regulate proliferation and apoptosis in response to stress [51]. This regulatory role may also be significant for mammary epithelial cells.

The downregulation of *DUSP26* could exacerbate tissue damage, leading to a reduction in milk quality, as evidenced by lower fat content, lower protein levels, and decreased casein concentration in SRLV-SP goats compared to SRLV-SN goats.

The results of this study suggest that reduced expression of *SCARA3* in SRLV-SP goats impairs its physiological functions, leading to the accumulation of reactive oxygen species (ROS) and cellular debris in the mammary gland, as reflected by an elevated somatic cell count. Functional analysis links *SCARA3* to oxidative stress-related pathways, where its downregulation appears to activate pro-inflammatory signaling cascades, including PTGS2 (COX-2). Consequently, decreased *SCARA3* expression may exacerbate mammary tissue damage and contribute to a decline in milk quality. Although direct studies on the role of APBB2 in mammary gland development are limited, its influence on the cell cycle and possibly on cell migration indicates that it may play a significant role in processes such as mammary gland cell morphogenesis and lactation.

Although the number of observations of milk traits per group was too limited to draw definitive conclusions regarding the impact of SRLV infection on milk yield and quality, the findings are consistent with our previous studies conducted on the same herd [52].

Although the downregulation of *PRLR* (prolactin receptor) identified by microarray was not confirmed as statistically significant in RT-qPCR analysis, this gene was retained in the results due to its well-documented role in mammary gland development, lactogenesis, and epithelial cell differentiation [53,54]. These processes are particularly critical during the first lactation, which was the focus of our study. The observed trend of decreased *PRLR* expression in SRLV-seropositive goats may suggest early dysregulation of hormonal signaling pathways in the mammary epithelium. Furthermore, PRLR has been functionally linked to growth factor-mediated signal transduction, and it modulates the expression of other genes downregulated in this study, such as APBB2. Although its role should be interpreted with caution, the presence of PRLR in the broader gene expression context may indicate an indirect effect of SRLV infection on hormone-mediated signaling and mammary tissue remodeling.

OR4F4 appeared downregulated in the microarray but could not be validated by RT-qPCR (Ct > 42 or undetected). This gene encodes an olfactory receptor GPCR, typically restricted to odorant detection in the olfactory epithelium. While ectopic OR expression in tissues like muscle, gut, and skin has been reported recently, no evidence links OR4F4 to mammary gland function, immune response, or viral infection in goats or ruminants. Therefore, to avoid speculative interpretation, OR4F4 was excluded from downstream functional analyses and figures [55].

This study has several limitations that should be considered when interpreting the results. First, the infecting genotype of small ruminant lentivirus (SRLV) in the seropositive goats was not determined, which limits the ability to link specific viral subtypes with the observed transcriptional alterations. SRLV genotypes are known to exhibit organ-specific tropism—for instance, genotype B2 has been associated with arthritic lesions in goats, and genotype A3 has been associated with neurological symptoms in sheep. Although genotype B is generally considered the most prevalent in Central Europe, recent findings by Kaba et al. (2022), conducted in the same goat herd as the present study, confirmed natural vertical transmission and seroconversion to SRLV genotype A, specifically subtype A17 [4]. This strongly supports the notion that genotype A circulates within this population and raises the possibility that genotype-specific viral tropism may contribute to the gene expression profiles observed in our study. Future studies combining transcriptomic data with viral genotyping are therefore warranted to investigate whether distinct SRLV genotypes differentially modulate host gene expression in mammary tissue.

Second, our transcriptomic analysis was performed on bulk milk somatic cells (MSCs), which comprise a heterogeneous cell population, predominantly mammary epithelial cells and leukocytes. The cellular composition of the samples was not determined, and as such, it remains unclear whether the observed gene expression changes reflect intrinsic regulation within epithelial cells or are influenced by the infiltration of immune cells in response to systemic or localized SRLV infection. However, our earlier study entitled “Gene expression profile in peripheral blood nuclear cells of small ruminant lentivirus-seropositive and seronegative dairy goats”, conducted in the same animals, revealed transcriptomic alterations in a completely different set of genes [19]. This suggests that the changes identified in the present study are most likely specific to the epithelial cells of the mammary gland. Nevertheless, future studies employing flow cytometry, cell sorting, or single-cell RNA sequencing approaches are needed to delineate cell-type-specific transcriptomic responses in SRLV-infected mammary tissue.

## 5. Conclusions

Downregulation of *DUSP26*, *APBB2*, and *SCARA3* expression in milk somatic cells (MSCs) of SRLV-seropositive goats during their first lactation suggests a potential involvement of these genes in the pathogenesis of Caprine Arthritis Encephalitis (CAE) within the mammary gland. Similarly, the downregulation of APBB2 may impair immune responses and increase apoptosis in udder cells, further decreasing productivity. The decreased expression of SCARA3 heightens oxidative stress, which damages udder tissues and negatively affects milk quality. Collectively, these molecular changes can result in lower milk production, poorer udder health, and weakened immunity, ultimately causing economic losses in dairy goat farming. Further studies using a much larger number of goats, both SRLV-SP and SRLV-SN, are necessary to clarify these mechanisms and guide targeted interventions for managing CAE.

## Figures and Tables

**Table 1:** Names, symbols, and NCBI accession numbers of genes and primers used in RT-qPCR verification of the microarray results.

Gene Name (Gene Symbol)	Primer (5′–3′)	Product Length	NCBI Accession Number	Source
*Capra hircus* dual-specificity phosphatase 26 (putative) (*DUSP26*)	F: GCACCCTTTCCTCAATGTCT	126 pb	XM_018042128.1	This study
	R: CGGTGGTTGTTGGCTATTTC			
*Capra hircus prolactin* receptor (*PRL-R*)	F: CTGTATCCTCCCACCAGTTC	177 pb	XM_013972804.2	This study
	R: TGTTGGTCCTCACTGTCATC			
*Capra hircus* scavenger receptor class A, member 3 (*SCARA3*)	F: AAATCTCCAAGGGCTGGATCT	234 pb	XM_018051874.1	This study
	R: ATCTGGTGAACGGAGAAAGAG			
*Capra hircus* amyloid beta (A4) precursor protein-binding, family B, member 2 (*APBB2*)	F: TTATGGCCGAACGGAAGAAT	223 pb	XM_018049396.1	This study
	R: TCCTTGCTAGAGGAGGTCAT			
*Capra hircus* olfactory receptor *OR4F4*	F: TTCTGTTCTTCGGACCATGT	164 pb	XM_005685436.2	This study
	R:TCTCAGCCGTCTCATTGC			
*Capra hircus* peptidylprolyl isomerase A (cyclophilin A) (*PPIA*)	F: GTCCCGAAGACAGCAGAAAA	151 pb	XM_018047035.1	This study—reference gene
	R: AGATGGACTTGCCACCAGTA			
Ribosomal protein large, P0 (*RPLP0*)	F: CAACCCTGAAGTGCTTGACAT	227 pb	NM_001012682.1	Reference gene [22]
	R: AGGCAGATGGATCAGCCA			
18S ribosomal RNA (*18S rRNA*)	F: CAAATTACCCACTCCCGACCC	114 pb	DQ_066896.1	Reference gene [22]
	R: AATGGATCCTCGCGGAAGG			

F: forward, R: reverse.

**Table 2:** Simple statistics of milk yield and composition in the morning milking of goats according to SRLV serological status.

	SRLV-SP			SRLV-SN		
	Mean	SD	CV [%]	Mean	SD	CV [%]
**MSCs (×1000)**	557.33	428.35	77	501.33	410.20	82
**Milk yield [kg]**	2.03	0.12	6	2.57	0.55	21
**Fat [%]**	2.59	0.07	3	2.77	0.20	7
**Protein [%]**	2.59	0.07	3	2.75	0.14	8
**Casein [%]**	1.76	0.07	4	2.02	0.14	7
**Lactose [%]**	4.77	0.10	2	4.78	0.19	4

MSCs—milk somatic cells; SRLV-SP—small ruminant lentivirus seropositive; SRLV-SN—small ruminant seronegative; SD—standard deviation; CV—the coefficient of variance.

**Table 3:** Microarray and RT-qPCR analysis of differentially expressed genes in MSCs of dairy goats at the peak of first lactation: a comparison of SRLV-SP and SRLV-SN goats.

Gene Name (Gene Symbol)	Direction of Expression Changes					
	Capra Hircus Microarray			RT-qPCR		
	Fold Change	Regulation	*p*-Value	Fold Change	Regulation	*p*-Value
PREDICTED: Capra hircus dual-specificity phosphatase 26 (putative) (*DUSP26*), *mRNA*	−11.38339	down	4.610555 × 10^−5^	−3.91994	down	0.01939
PREDICTED: Capra hircus prolactin receptor *(PRLR)*	−5.3226624	down	8.543044 × 10^−5^	−1.25194	down	0.24871
PREDICTED: Capra hircus scavenger receptor class A, member 3 (*SCARA3*)	−3.5556233	down	8.145998 × 10^−5^	−3.38698	down	0.02875
PREDICTED: Capra hircus amyloid beta (A4) precursor protein-binding, family B, member 2 (*APBB2*)	−2.9709337	down	5.4867196 × 10^−5^	−1.76949	down	0.039
PREDICTED: Capra hircus olfactory receptor 4F4 (*LOC102187321*), *mRNA*	−3.3447337	down	5.5302753 × 10^−5^	nd	nd	-

## Data Availability

The data supporting the findings of this study are publicly available in the NCBI Gene Expression Omnibus (GEO) database under the accession number [GSE299223], available at https://www.ncbi.nlm.nih.gov/geo/query/acc.cgi?acc=GSE299223 (accessed on 2 July 2025).

## Supplementary Materials

The following supporting information can be downloaded at https://www.ncbi.nlm.nih.gov/geo/query/acc.cgi?acc=GSE299223 (accessed on 2 July 2025).
